# Role of Neoplastic Markers in Pancreatic Adenocarcinoma

**DOI:** 10.3390/jcm11216509

**Published:** 2022-11-02

**Authors:** Alessandro Coppola, Tommaso Farolfi, Vincenzo La Vaccara, Roberto Cammarata, Damiano Caputo

**Affiliations:** 1 Department of Surgery, Sapienza Università di Roma, 00185 Rome, Italy; 2 General Surgery, Università Campus Bio-Medico di Roma, 00128 Rome, Italy; 3 General Surgery, Fondazione Policlinico Universitario Campus Bio-Medico, 00128 Rome, Italy

Pancreatic ductal adenocarcinoma (PDAC) is considered one of the “Big Five” lethal cancers, which include lung, bowel, breast and prostate cancer. PDAC is set to be a second-place candidate for the most cancer-related deaths [1].

The poor survival rates in PDAC patients can be explained both by the aggressive behaviour of the disease and by the lack of reliable tools for its early diagnosis.

In 80–85% of cases, PDAC is diagnosed in the advanced or metastatic stages, and only a minority of patients are eligible for radical surgery, which still represents the gold standard treatment [2].

As recently reported by Klaiber et al., early diagnosis would allow doctors to identify PDAC at an early stage, increasing the 5-year survival rate [3].

Following the guidelines of the Food and Drug Administration, despite its low specificity (82%) and sensitivity (79%), CA 19.9 is the only available neoplastic marker recognized for the clinical management of PDAC [4].

Unfortunately, CA 19.9 is not without limitations: first, about 10% of the population does not secrete CA 19.9, causing a large number of false-negatives results; second, cholestatic jaundice could increase CA 19.9 values and may also occur in cases of non-malignant lesions [5].

An aberrant pathway of disialyl Lewis (a) production is responsible for the formation of CA 19.9 (a sialyl Lewis (a) antigen).

The role of this molecule is to maintain immunological homeostasis, and it functions as a ligand for E-selectin on endothelial cells; this explains why PDAC secreting CA 19.9 usually shows biological aggressiveness and why non-secretor PDAC patients have better prognoses [6,7,8].

For early diagnosis of PDAC, due to the weak and limited evidence reported in the literature, the role of CA 19.9 is still not defined or recognized in clinical practice [9].

Today, a well-established field of clinical application for CA 19.9 is the monitoring of radio- and chemotherapy efficacy in patients with advanced PDAC [10].

As demonstrated by Robert et al. in a retrospective analysis of 342 patients with metastatic PDAC treated with FOLFIRINOX, a CA19.9 decrease after therapy of more than 20% is a prognostic factor for both overall survival (OS) and progression-free survival [11].

In resectable-PDAC patients undergoing surgery, CA 19.9 has similarly been shown to be useful in monitoring during adjuvant treatment to predict disease recurrence [12].

As previously reported, preoperative serum levels of CA 19.9 have limited utility in the diagnosis of PDAC, but in recent years, this marker has become increasingly important in determining the prognosis and being part of the decisional therapeutic process of this malignancy [13].

More in detail, Ferrone et al., in 2006, demonstrated that a pre-operative cut-off value of CA 19.9 under or above 1000 U/mL reflected a statistically significant longer median OS when the two groups were compared, with survival changing from 28 months to 12 months [14].

In 2019, a multicenter Italian Study showed that preoperative CA 19.9 serum levels of 100 U/mL or above were associated with worse survival outcomes. They also showed a significant association between CA 19.9 and pathological nodal involvement [15].

Similarly, the ability of CA 19.9 values to predict early local recurrence is also demonstrated by numerous studies. Fiore et al., using a cohort of 120 PADC patients, analyzed the pre-operative CA 19.9 value in combination with the PET/CT maximum SUV level.

Notably, in this report, CA 19.9 values were also significantly associated with oncological outcomes such as early local recurrence and overall survival. In detail, a CA 19.9 value above 698 U/mL in pre-operatory measurement is associated with a six-times higher risk of early recurrence [16].

In the past year, the role of pre-operative CA 19.9 in predicting lymph-node positivity (N+) after resection has been explored extensively; in a large series of patients, the Heidelberg group demonstrated that the presence of positive lymph nodes represents one of the most significant prognostic factor for long-term oncological outcomes after upfront surgery in patients with PDAC [17]. 

CA 19.9 has been used in combination with radiological findings (CT or PET-CT) to improve their sensibility and specificity in predicting N+ [18,19].

In 2021, Coppola et al. confirmed Mattiucci’s findings in a single-center retrospective analysis whereby a cohort of 165 patients with resectable PDAC underwent pancreatic surgery. In this paper, values of CA 19.9 > 37 U/mL were able to predict the presence of positive lymph nodes in the final pathology report. Notably, in the presence of hypoalbuminemia, CA 19.9 was not able to predict lymph nodal involvement [20].

Takahashi et al. confirmed the previous findings in a larger retrospective study. From an analysis of more than four hundred patients with resectable disease, they demonstrated that the overall survival of patients with pre-operative CA 19.9 values greater than 120 U/mL is similar to that of patients with anatomical borderline resectable PDAC. In addition, a reduction in marker levels after neoadjuvant therapies was demonstrated to be a predictor of improved oncological and survival outcomes [21].

Therefore, considering the above-mentioned studies, new perspectives on the role of CA 19.9 must be explored. This marker is becoming crucial in PDAC preoperative staging. As such, the idea of biological resectability in pancreatic cancers where CA 19.9 may represent a fundamental, feasible and inexpensive tool to establish more appropriate therapeutic strategies is gaining momentum; this is particularly true considering the encouraging results of Japanese trials comparing oncological outcomes after neoadjuvant treatments in borderline and resectable patients with PDAC [22].

The second most common neoplastic marker adopted in clinical practice is the serum carcinoembryonic antigen (CEA); its levels have increased by between 30% and 60% in pancreatic cancer [23].

This glycoprotein was detected for the first time in patient with bowel adenocarcinoma [24].

CEA has been proposed as an alternative marker to use in CA 19.9 no-secretor patients; however, the relationship between CEA and PDAC still remains unclear.

In 2016, Imaoka and colleagues published a retrospective study in a series of 433 patients, demonstrating that in patients with metastatic disease, CEA levels represent a survival prognostic factor. In detail, 6.8-month OS was observed in patients with CEA > 5 ng/mL, unlike the 10.3 months in patients with CEA under the previous cut-off [25].

Considering the limited use of CEA alone, many authors have proposed the use of this serum marker in combination with CA 19.9 and other markers to improve its diagnostic power.

In 2008, the Hellenic Group of Oncology found, using multivariate analysis, that higher levels of neoplastic marker, CEA and CA19.9 had a negative impact on the prognosis and survival of patients with unresectable and metastatic pancreatic cancer [26].

More recently, an Austrian study found that the prognostic efficacy of the combination of CEA and CA19.9 was significantly higher compared with single tumor markers [27].

In 2015, Liang et al. investigated the prognostic role of a panel of three markers: CA 19.9, CEA and CA 125.

A series of over 1000 patients underwent pancreatectomy at two independent high-volume institutions, and it was found that the association between CA 19.9, CEA and CA 125 was able to predict poor surgical outcomes with the lowest median survival (5.1 months vs. 23.0 months in the control group). In addition, the authors suggest the possibility of a higher prevalence of early distant metastasis after radical surgical treatments in patients with pre-operative elevated neoplastic markers [28].

Other serum markers such as CA 50 and CA 125 have been widely used, alone and in combination with each other, in order to find a useful tool both for diagnostics and prognostics in PDAC patients with poor results.

In conclusion, the above-mentioned markers seem to be a useful tool in the diagnosis of PDAC, as well as in evaluating its response to neoadjuvant or adjuvant treatment, disease progression and further prognosis.

Unfortunately, there is still not enough evidence to guide decision-making for the treatment of patients with adenocarcinoma of the pancreas.

Future studies with larger cohorts of patients and, ideally, in prospective randomized settings could improve our knowledge on the efficacy of these neoplastic markers, alone or in combination with new technologies such as nanoparticles and/or circulating tumor cells.
